# Antibody and Protein Profiles in Glaucoma: Screening of Biomarkers and Identification of Signaling Pathways

**DOI:** 10.3390/biology10121296

**Published:** 2021-12-08

**Authors:** Nadine Auler, Henrik Tonner, Norbert Pfeiffer, Franz H. Grus

**Affiliations:** Department of Experimental and Translational Ophthalmology, University Medical Center, Johannes Gutenberg University, 55131 Mainz, Germany; nauler@eye-research.org (N.A.); htonner@eye-research.org (H.T.); Norbert.Pfeiffer@unimedizin-mainz.de (N.P.)

**Keywords:** autoantibodies, glaucoma, neuroprotection, diagnosis, microarray, mass spectrometry, immunology, molecular signaling pathways, experimental glaucoma models

## Abstract

**Simple Summary:**

Glaucoma is a chronic eye disease that is one of the leading causes of blindness worldwide. Currently, the only therapeutic option is to lower intraocular pressure. The onset of the disease is often delayed because patients do not notice visual impairment until very late, which is why glaucoma is also known as “the silent thief of sight”. Therefore, early detection and definition of specific markers, the so-called biomarkers, are immensely important. For the methodical implementation, high-throughput methods and omic-based methods came more and more into focus. Thus, interesting targets for possible biomarkers were already suggested by clinical research and basic research, respectively. This review article aims to join the findings of the two disciplines by collecting overlaps as well as differences in various clinical studies and to shed light on promising candidates concerning findings from basic research, facilitating conclusions on possible therapy options.

**Abstract:**

Glaucoma represents a group of chronic neurodegenerative diseases, constituting the second leading cause of blindness worldwide. To date, chronically elevated intraocular pressure has been identified as the main risk factor and the only treatable symptom. However, there is increasing evidence in the recent literature that IOP-independent molecular mechanisms also play an important role in the progression of the disease. In recent years, it has become increasingly clear that glaucoma has an autoimmune component. The main focus nowadays is elucidating glaucoma pathogenesis, finding early diagnostic options and new therapeutic approaches. This review article summarizes the impact of different antibodies and proteins associated with glaucoma that can be detected for example by microarray and mass spectrometric analyzes, which (i) provide information about expression profiles and associated molecular signaling pathways, (ii) can possibly be used as a diagnostic tool in future and, (iii) can identify possible targets for therapeutic approaches.

## 1. Introduction

Glaucoma is defined by the European Glaucoma Society as a chronic progressive optic neuropathy, with morphological changes at the optic nerve head and retinal nerve fiber layer associated with retinal ganglion cell death and visual field loss. It is the second leading cause of blindness worldwide and the most common reason for irreversible blindness [1]. To date, approximately 53 million people worldwide are affected, although an increase to 80 million affected persons is proposed by 2040 [2]. Glaucoma comprises a heterogeneous group of chronic ocular diseases, which are divided into juvenile and adult forms, and the adult forms are further differentiated into primary and secondary forms of glaucoma. The primary forms are additionally subdivided into primary open-angle glaucoma (POAG) and acute or chronic closed-angle glaucoma. POAG referred to in this review is characterized by increased outflow resistance in the area of the trabecular meshwork cells in the anterior chamber of the eye, resulting in decreased aqueous humor (AH) outflow [1]. Under physiological conditions, trabecular meshwork cells have a fibroblast-like character that provides stable contraction and thus modulates AH outflow. In POAG patients, there is an accumulation of extracellular matrix components, as well as destabilization of the actin cytoskeleton, leading to dysregulation of AH outflow, and resulting in an elevated intraocular pressure (IOP) [3,4]. The increase in IOP causes mechanical stress and induces the production of reactive oxygen species (ROS) stimulated by changes in mitochondria [5]. High ROS levels enhance autophagy and cell dysfunction, are associated with inflammatory responses, and are able to activate apoptotic pathways and cell death resulting in loss of vision [6,7,8]. Although elevated IOP is considered the highest risk factor along with age, ethnicity or myopia, many glaucoma patients do not have an elevated IOP and are therefore classified as normal-tension glaucoma (NTG) patients. In addition to these risk factors, pseudoexfoliation syndrome (PEX), an age-related disease of the extracellular matrix, is also considered a risk factor for developing glaucoma. In NTG patients, it is assumed that destabilization of the lamina cribrosa leads to destabilization of the ganglion cell axons and thus to a dysregulation of the ganglion cell nutrition. The risk factors age and increased IOP correlate directly with increased reactive oxygen species and mitochondrial dysfunction [9], resulting in a metabolic missupply, and consequent death of ganglion cells. In addition to metabolic alterations, downregulation of certain genes, as well as neurodegenerative and immunologic responses, also play a role in glaucoma [10,11], making it a multifactorial disease that can be considered both a neurologic but also possibly an autoimmune disease [12,13,14,15]. However, so far, a reduction of IOP as the only treatment option can only slow down disease progression but fails to put it to halt. This problem of missing causative treatment even intensifies as the diagnosis is usually made at advanced stages of disease when ganglion cells have already been lost. This is reflected in the fact that approximately 26% of all patients do not experience any symptoms at all [16] and it is even estimated that undiagnosed glaucoma is an invisible size, which probably includes again the same number as diagnosed glaucoma [17,18]. This makes it necessary to find a way to diagnose glaucoma early before the nerve fibers are damaged. Furthermore, the multifactorial nature of the disease shows that other therapeutic approaches besides IOP reduction are necessary to stop progression of glaucoma. To address these two issues, proteomic studies are nowadays performed to screen altered protein expression patterns to find biomarkers that could serve as diagnostic markers, progression determination or even as potential therapeutic targets, which will be discussed in this review.

## 2. Diagnosis of Glaucoma and Screening for Potential Biomarkers

To date, there are only a few tests to diagnose glaucoma and no procedures for comprehensive population-wide periodic screenings. The main method used for initial diagnosis is ophthalmoscopy to assess an enlargement of optic nerve excavation, nerve fiber layer thinning, or hemorrhages at the optic nerve disc. Additionally, the measurement of IOP by tonometry, the assessment of the central cornea thickness by pachymetry, and examinations of the chamber angle by gonioscopy are necessary and can give information about the pathogenesis. Because IOP is the only modifiable risk factor to date, a diurnal intra-individual pressure profile is made, with the main objective of reducing IOP by medications or surgery. IOP lowering is known to reduce, but often not prevent, glaucoma progression. Therefore, the progression of the disease must be examined perimetrically in close-meshed steps via visual field assessment. The progression of visual field loss is difficult to detect because of the compliance of patients and the compensation by binocular vision.

Identification of biomarkers could allow the early detection of diseases. The U.S. Food and Drug Administration (FDA) defines a biomarker, depending on its use, as a diagnostic marker, to assess the severity of disease, predict progression, or predict the response to treatment. What they have in common is that a biomarker must be an objective, quantifiable, or qualifiable biological indicator that is used as a characteristic reference for an event, condition, or process. Molecular biomarkers are already widely used for diagnosis and prediction of treatment response, for example, mutations in the BRCA genes are implicated in the pathogenesis of familial breast and ovarian cancer and are used as genomic biomarkers [19]. Biomarkers also play a crucial role in the diagnosis and prediction of eye diseases. Vascular endothelial growth factor (VEGF), a factor associated with vascular diseases, was found as a predictor of severity for diabetic retinopathy progression after vitrectomy in two studies [20,21].

A link between glaucoma and genetics was established in the early 1980s. Although POAG rarely follows the Mendelian genetic, it is nevertheless not surprising that genetic markers are used as biomarkers in hereditary forms of glaucoma. For example, mutations in the genes encoding myocilin [22], and neurotrophin 4 [23] are considered markers for adult-onset glaucoma, while mutations in the genes encoding optineurin [24], and WD repeat-containing protein 36 [25] have no direct link to the pathogenesis of glaucoma, but suggest increased susceptibility of ganglion cells, especially in normal-tension glaucoma. Such mutations are associated only with a small percentage of adult POAG [26], highlighting that genetic markers alone are insufficient to be classified as biomarkers and to find an overarching early diagnosis for patients affected by glaucoma. Altered protein concentrations have previously been measured with radioimmunoassays or protein assays, such as increased endothelin-1 levels in plasma [27] or increased gelatinase A activity in AH from POAG patients [28]. These methods have the disadvantage that only single proteins could be targeted and, in addition, biomarker definitions are not achieved.

Today the “Omics” are very prominent to study biomarkers. Omic-based examinations were used as a diagnostic tool before the term was introduced. In the 1980s and 1990s, proteins were studied for example by gel electrophoresis or isoelectric focusing and were already used for diagnosing metabolic diseases, Crohn’s disease, or multiple sclerosis [29]. The main limitations of these methods for the identification of biomarkers are the lack of quantifiability and the missing measurement of complex multi-factorial protein changes. Omic-based methods approach these problems via technological improvements in sequencing, mass spectrometry, and the further development in bioinformatics [30], giving the chance for high-throughput detection and analysis in protein (proteomics), metabolite (metabolomics), lipid (lipidomics) or gene (genomics/epigenomics) alterations in several body tissues and liquids. In the next section, we will focus on glaucoma biomarkers on the protein level, as proteomics has increasingly gotten into focus for biomarker research in recent decades. Proteomics can be used to measure protein abundance in different tissues and fluids. For glaucoma, retina samples, as well as vitreous or AH samples, are used and can give insights into the pathogenesis of glaucoma directly at the site of damage. The disadvantage of these tissues is that they are not accessible non-invasively and thereby disqualify for routine diagnostic purposes. Retinas can only be sampled from donor’s eyes, while vitreous and aqueous humor can additionally be taken by vitrectomy, usually also frequently during cataract surgery or trabeculectomy. Furthermore, the flaw for proteomic studies of these materials often is that no matched control samples are available, as such an intervention would not be ethical in healthy individuals. Thus, these tissue samples are more useful for investigating glaucoma pathogenesis not diagnosis. Therefore, it is essential to examine protein distribution in different body fluids such as blood or tear samples for early diagnosis of otherwise healthy people and to find marker compositions that are also significantly measurable. Tear fluid can be analyzed for protein alteration, but often already shows changed distributions when glaucoma patients are treated with medication.

In this review, we will discuss proteins overlapping from profiles analyzed by various methods and different researchers. Consistent protein profiles, discussed in the following section, give a higher accuracy compared to single-identifications and must serve as a benchmark.

### 2.1. Proteins Involved in Cytoskeleton Organization

Most frequently, a down-regulation of crystallins is measured in different samples of glaucomatous patients or donors. Crystallins are structural proteins in the lens and other eye tissues and can be divided into two subfamilies, α- and β-/γ-crystallins.

The α-crystallins, classified into αA and αB, are members of the small heat shock protein family. The αA-crystallins are restricted to the lens where they can bind non-native proteins or β-/γ-crystallins to prevent the aggregation and insolubilisation of these proteins giving the lens their refractoriness [31]. αB-crystallins are also expressed in other ocular tissues like retina, cornea, optic nerve, astrocytes, and Müller cells. αB-crystallins can modulate the transcription factor nuclear factor ‘kappa-light-chain-enhancer’ of activated B-cells (NFκB) to protect cells from the tumor necrosis factor α (TNF-α) cytotoxicity [32], working as a protective protein with chaperone activity. The β/γ-crystallins form their superfamily characterized by a distinct structure called Greek key motif. The β crystallins can convert into complex oligomers while the γ-crystallins are present as monomers, whereas in the mammalian retina crystallins of all superfamilies are expressed in the lens, in the human lens mainly γ-crystallins are measurable.

**Figure 1:**
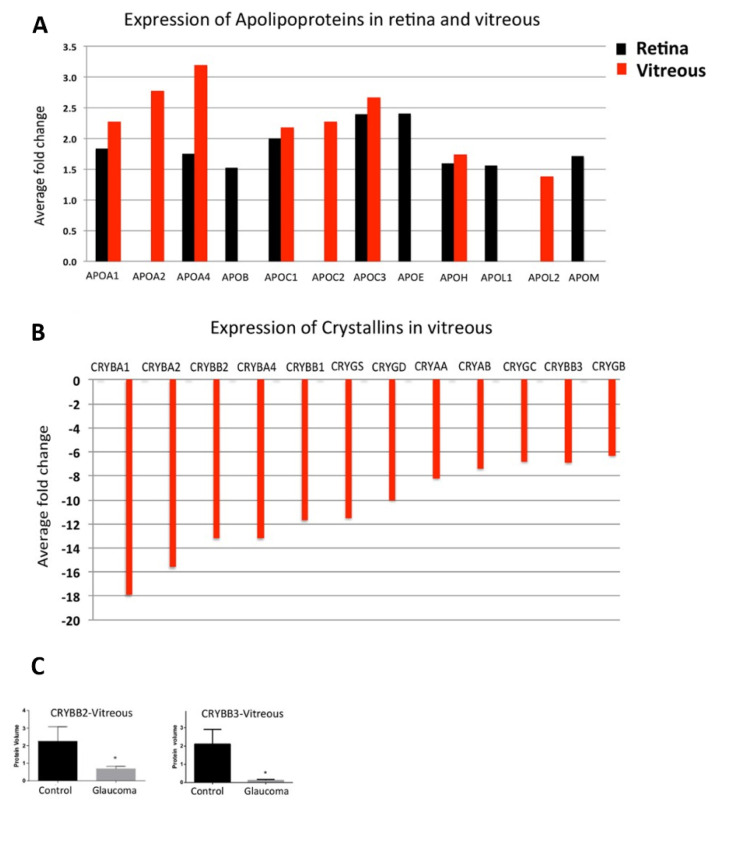
Upregulation of Apolipoproteins and down regulation of Crystallin and GSTs in glaucoma. (**A**) A bar graph representing the expression pattern of 12 differentially expressed Apolipoproteins identified in retina and/or vitreous (*p*-value ≤ 0.05 and ≥1.3-fold change, *n* = 10). (**B**) A bar graph representing the relative abundance of 12 differentially expressed crystallin proteins identified in vitreous (*p*-value ≤ 0.05 and ≤0.77-fold change, *n* = 10). (**C**) Western blotting analysis for measuring the relative protein expression level of CRYBB2 and CRYBB3 in vitreous (*n* = 10) of glaucoma and control samples. GAPDH was used as the loading control. The bar graphs indicate average densitometry measurements (ImageJ software) (*n* = 10, average ± SD, * *p*-value < 0.05). Black bars represent control, while the light grey bars represent glaucoma. The figure is modified based on the figure published by Mirzaei, et al. 2017 [33]. This work is open access. The Copyright is available online: http://creativecommons.org/licenses/by/4.0/ (accessed on 7 December 2021).

Different protein profiles in glaucomatous retina, vitreous, and AH are measured by proteomics. Mirzaei et al. 2017 [33] analysed vitreous and retina samples of glaucomatous donor eyes in comparison to healthy donor eyes by LC-MS/MS and found a down-regulation of 12 members of the crystallins exclusively in the vitreous (Figure 1, Table 1). Some other studies supported these measurements. αA-crystallin is also found to be down-regulated in the AH of glaucoma donors [34], while βA1-crystallin and βB1-crystallin were measured to be down-regulated in the glaucomatous retina [35]. Contrary to the measurements in the vitreous of glaucoma patients by Mirzaei et al., αB-crystallin was found to be up-regulated in the glaucomatous retina [35]. There could be different explanations for this: First, the glaucoma phenotype could be different. While Mirzaei et al., 2017 [33] specify that retinas were collected from donors with open-angle glaucoma, Funke et al., 2016 [35] cannot provide any information on the glaucoma phenotype. Second, the retina of the donors was separated from other eye tissues after death, thus contamination, especially with lens tissue, cannot be excluded. In two different experimental rat glaucoma models (both in Spraque Dawley rats) with chronic elevated IOP, once by microbead injection (MB) and measurement of protein changes after eight weeks [36] and the other through episcleral vein occlusion (EVO) and measurement after five weeks. A down-regulation of many crystallins of all subfamilies could also be detected here in retinal tissue [37]. In comparison, Piri et al., 2007 [38] induced chronically elevated IOP in rats by Trabecular laser photocoagulation (TLP) and analysed retinal protein levels as well as mRNA levels after two and five weeks after IOP elevation. The mRNA and the protein level of crystallins have been found down-regulated after two weeks, while after five weeks the mRNA level returns to the level of the control group. In contrast, the protein level of the crystallins after week five was higher than after week two but did not reach the quantity of the control crystallins.

The authors give three possible explanations for the difference between mRNA and protein level, (1) the crystallin expression is modified post-transcriptionally, (2) the protein translation is delayed or (3) the turnover rate of crystallins is enlarged. In an age-related IOP-independent model induced by optic nerve crush (ONC) in mice, a downregulation of different crystallins has also been found in experimental retinas [39]. In general, down-regulation of crystallins in glaucoma is rather unexpected, as they are enhanced in aging as well as in other ocular diseases such as cataract [40,41], age-related macular degeneration (AMD) [42], or diabetic retinopathy (DR) [43] as well as in neurodegenerative disorders like Alzheimer disease (AD) [44], Alexander’s disease [45] or Parkinson’s disease (PD) [44]. Furthermore, in experimental animal models, overexpression of crystallins was observed for autoimmune uveitis [46], or multiple sclerosis [47]. In glaucomatous vitreous, the crystallins are clustered with other proteins involved in cholesterol transport or apoptosis investigated by Reactome pathway analysis, suggesting that down-regulation in glaucoma increases the susceptibility of ganglion cells to cytotoxic stress [33]. Interestingly, the studies mentioned above show that in glaucoma a down-regulation of crystallins can be found in different tissues. Deviations from these measurements demonstrate that further studies are necessary to validate the correlation of crystallin down-regulation with glaucoma pathogenesis. A down-regulation of crystallins is not glaucoma specific as they could also be observed in the AH of patients with high myopia [48], although it should be noted that myopia is a risk factor for the development of glaucoma [49].

Additionally, other proteins involved in cytoskeleton organization and protein folding are found to be differentially expressed in the glaucomatous retina. Three types of tropomyosins (TPM1, TPM3, and TPM4), the RAS GTPase-activating-like protein IQGAP2 (IQGAP2), and the neuroblast differentiation-associated protein AHNAK (AHNAK) were found to be down-regulated in human and rat retina [36]. Contrary, IQGAP2 protein level was found to be highly up-regulated in EVO-induced rat models [37]. Concerning the possibility that glaucoma is an autoimmune disease, it is worthy to note that in patients with Sjögrens syndrome, a chronic autoimmune disease, an up-regulation of circulating IQGAP2 RNA was identified, while the IQGAP2 protein level was not differentially expressed [58]. Furthermore, IQGAP2 protein level is found to be down-regulated in patients with ovarian cancer and is negatively correlated with progression and survival of patients thus implicated as a biomarker for ovarian cancer [59]. The authors demonstrated that IQGAP2 can suppress the transcriptional activity of β-catenin, a downstream protein in Wnt signaling, which leads to an inhibition of cancer cell epithelial-mesenchymal transition, migration, and invasion.

Even proteins involved in the Wnt signaling pathway were found differentially expressed in the AH of glaucoma patients. Dickkopf (DKK), wingless-related integration inhibitory factor 1 (WIF1), and pigment epithelium-derived factor (PEDF/SERPINF1) were significantly up-regulated, known as Wnt antagonists [50]. The inhibition of the Wnt signaling pathway is implicated to be involved in an elevated IOP through an increased TM cell stiffness [60] as well as in dysregulation of dendritic outgrowth, axon remodeling, and function of neurons characteristic for glaucoma [61], thus overexpression of IQGAP2, as published for EVO model, appears explainable.

In several other neurodegenerative diseases, diminished Wnt-β-catenin signaling is observed associated with pathological progression, such as AD or PD [62,63]. Therefore, further investigation of the differentially expressed proteins should take place to determine whether they can be used as diagnostic or progression markers.

### 2.2. SERPIN Gene Family

PEDF, a member of the serine protease inhibitor superfamily (SERPINs), showed increased levels in the AH of glaucoma patients compared to healthy subjects, while it is a prominent down-regulated marker in different tissues in neovascular eye diseases such as AMD [64] or DR [65]. Comparable to glaucoma, an enriched level of PEDF could be detected in tears of dry eye patients [66]. This up-regulation is an effect that can be explained by inflammatory processes after the onset of glaucoma. PEDF, a neurotrophin secreted by Müller cells and highly involved in retinal inflammation, has an anti-angiogenic but also a neuroprotective potential. Secreted PEDF can activate NFκB, which controls the expression of anti-apoptotic proteins such Bcl-2 or Bcl-x and simultaneously leads to an up-regulation of neuroprotective mediators like BDNF and NGF [67,68,69]. The up-regulation of these mediators in turn suppresses downstream apoptotic cascades promoting RGC survival. Aside, while the activation of neuronal NFκB is involved in neuron survival, plasticity and synapse formation [70,71], the transcriptional activity of glial-driven NFκB plays an important role in neuroinflammatory priocesses in glaucoma [72,73]. Nevertheless, reduced levels of PEDF and consequently reduced inhibition of angiogenesis could serve as a marker for neovascularization but elevated levels fail to be classified as a diagnostic or progression marker for glaucoma in particular. Still, it provides good approaches for therapeutic options.

Furthermore, several other proteins expressed by the SERPIN genes, named by their function as serine protease inhibitors, are differentially regulated in glaucoma patients. α-1 antitrypsin (SERPINA1/ATT), α-1 antichymotrypsin (SERPINA3/AACT), corticosteroid-binding globulin (SERPINA6/CBG), thyroxine-binding globulin (SERPINA7/TBG), angiotensinogen (SERPINA8/AGT), and α-2 antiplasmin (SERPINF2/A2AP) were overrepresented in the AH of glaucoma donor eyes, as measured by mass spectrometry [34]. All of them are implicated in oxidative stress and inflammatory processes.

Interestingly, neuroserpin encoded by SERPINI1, primarily expressed in neuronal cells, was down-regulated in the AH of glaucoma patients, analyzed by mass spectrometry [34]. Neuroserpin inhibits extracellular plasmin as well as plasmin activators in neuronal tissues, involved in brain development and maintenance through regulating neuronal plasticity and survival [57]. In contrast, Gupta et al., 2017 [52] could not find any different expression levels of neuroserpin or its target plasmin in the vitreous, retina, or optic nerve head of glaucoma patients in comparison to healthy subjects analyzed by Western Blot. These different outcomes could be explained by various aspects: (1) possibly, mass spectrometry is more sensitive to determine differentially expressed protein levels than Western Blot analysis, (2) while the AH samples were collected from living patients and were compared to cataract patients, the samples of the vitreous, retina and optic nerve head were collected from human cadaver eyes and compared to healthy subjects, (3) Keaslin et al. [34], classified the patients as POAG, while Gupta et al., [52] provided no information about glaucoma phenotype. That neuroserpin was down-regulated in AH, but not in other glaucomatous tissues seems implausible, as in healthy tissues protein levels in the retina and ONH were significantly higher than in the vitreous sample [52].

Plasmin is also involved in the complement system, known to cleave complement C5 at the same rate as canonical C5 convertase [74]. However, the C5 protein level was not changed in the glaucomatous retina [55]. This is not yet an indication that neuroserpin expression is diminished, since plasmin is additionally regulated by α-2 antiplasmin, which, as already mentioned, is overexpressed in the AH of glaucoma patients.

However, regardless of whether neuroserpin is down-regulated, plasmin inhibition is reduced, which in glaucoma strongly correlates with increased degradation of ECM (extracellular matrix) proteins in the retina [52]. Restoration of ECM homeostasis in TM cells and the retina could be a therapeutical approach to prevent disease progression.

Therefore, further studies are necessary to possibly detect neuroserpin as a progression marker for glaucoma. Although polymerization and aggregation of neuroserpin are correlated with the so-called serpinopathies, including Familial Encephalopathy with Neuroserpin Inclusion Bodies (FENIB) and up-regulation in AD are more likely, also down-regulation of serine protease inhibitor in multiple sclerosis is discussed [75].

### 2.3. Apolipoproteins

Another field of differentially regulated proteins in glaucoma patients are apolipoproteins. Mirzaei et al., 2017 [33] described a direct link between apolipoprotein expression and glaucoma, giving a prediction of glaucomatous effects in the future. They found significant up-regulations of different apolipoproteins in the retina and vitreous of glaucomatous donor eyes (Figure 1, Table 1). This correlates well with the results obtained from proteomic analyses of the AH of glaucoma patients, which also revealed increased levels [34,50,53].

APOA1 plays an important role in the regulation of reverse cholesterol transport and is involved in the protection of the vascular system by preventing cholesterol deposition due to interactions with HDL. A similar effect is also thought to occur in AD, where APOA1 may interact with Aβ to protect neurons from the toxic effects of Aβ deposition [76]. In contrast, lower levels of APOA1 led to lower cholesterol homeostasis and function in the brain, as in PD which suggests it is a potential biomarker for PD [77,78].

APOD is also very important for cholesterol transport mediating the interaction between HDL and LDL, but is also involved in mechanisms protecting against oxidative stress, is essential for the maintenance of nerve function, and regulates the number of macrophages recruited to injury sites [79,80]. APOD is generally overexpressed in neurodegenerative diseases, whereas in PEX syndrome, an age-related disease of the extracellular matrix, a significant underrepresentation of APOD in the AH could be detected [81]. Thus, the authors suggested APOD as a potential biomarker for PEX. However, it is also relevant for glaucoma, as PEX syndrome is a risk factor for the development of glaucoma.

In glaucoma, overexpression of APOE is restricted to the retina and not measurable in the vitreous [33], demonstrating a direct link to neurons. Human APOE is expressed in three isoforms: APOE2, APOE3, and APOE4. The APOE4 allele is known to be a genetic risk factor for the development of AD [82], suggested also for AMD [83]. APOE4 in the CNS is thought to have a direct toxic effect on neurons, through impaired neurite growth and synapse formation, cytoskeleton malfunction, or mitochondrial dysfunction, and is characteristic of reduced amyloid β clearance in AD [84]. Interestingly, individuals expressing the APOE2 allele have a lower risk of developing AD than individuals expressing the APOE4 allele [82]. Regarding prognosis, it would also be interesting to investigate the involvement of the APOE alleles in glaucoma.

To be considered as biomarkers for the diagnosis of glaucoma or even as progression markers, all the proteins summarized so far must fulfill one additional characteristic. They must also be detectable in a validatable manner in the body fluids of glaucoma patients.

Gonzalez-Iglesias et al., 2014 [51] detected different biomarker candidates with 2D-DIGE analyzed by MALDI-MS and nano LC-MS/MS and identified 35 different regulated proteins between glaucoma serum samples and healthy controls.

APOA1 and APOA4 were significantly 1.3 and 2.7 fold up-regulated in serum samples of glaucoma patients in comparison to controls. They additionally performed an individual biomarker power discrimination with newly recruited glaucoma patients and found APOA4 yielded the best performance correctly discriminating 97% of glaucoma patients from healthy subjects with a sensitivity of 100% and a specificity of 95% (Table 2). Additionally, a distinction of 81% of glaucoma patients from patients with PEX syndrome could be detected. APOA1, on the other hand, had only discriminatory power to identify 76% of all glaucoma patients from healthy subjects. Two other interesting biomarkers mentioned above are C3 and α-1-antitrypsin, which were also found to be significantly up-regulated in the serum of glaucoma patients [51]. Both had an identification rate of more than 80% but with lower sensitivity and specificity than APOA4. This represents APOA4 as a strong biomarker candidate to identify glaucoma patients in comparison to healthy subjects; however, it is not known whether it could distinguish other neurodegenerative diseases.

### 2.4. Complement System

Proteomic studies show that other components than C5 of the complement cascade are dysregulated. The complement system is involved in innate and adaptive immunity and dysregulation is therefore associated with some autoimmune diseases. It can be activated by three signaling pathways: the classical, the lectin, and the alternative pathway. The understanding of functional complement activity and different regulation of proteins are already being used to predict the presence of certain diseases using hemolytic assays. For example, a low measured CH50, reciprocal for 50% hemolysis, low levels of C3 and C4, and a normal factor B value, are indicative for classical pathway activation implicated in autoimmune disorders like Sjogren’s syndrome, systemic lupus erythematosis (SLE), or rheumatoid arthritis [85]. In the AH of glaucoma patients, complement component C1q, C8 β chain, C9, and V-set immunoglobulin domain-containing protein 4, were measured overexpressed [34]. This correlates with up-regulated levels of C1, C8, and C9, as well as MASP1 and MASP2 in retinal tissues from glaucoma donor eyes [54]. Additionally, the major component C3, involved in all three signaling pathways, could be measured up-regulated in the retina and AH of glaucoma patients [50,55]. In a recent study, an increased ratio of C3 to C3a was found in both aqueous humor and serum of progressive POAG patients compared to patients with stable POAG and cataract patients who served as controls [86]. It is striking that in serum and aqueous humor, the C3 level remained approximately comparable between the groups. Accordingly, the ratio increased mainly due to the change in the C3a level. The changes in C3a expression could only be detected in the progressive POAG group and not in the stable POAG group. The authors concluded that the C3a/C3 ratio correlates with disease status and that the level of this ratio describes a measure of the rate of disease progression. Supporting an involvement of the complement system in glaucoma, in IOP-independent experimental autoimmune glaucoma model (EAG) an increase of C3 positive cells and an increase of C3 mRNA level could be found 14 days after induction, while the C5 level remained unchanged [56]. During acute or chronic inflammation, overexpression of C3 is known to occur not only in glaucoma but also in other eye diseases like AMD [87] or DR [88]. Therefore, classical components of the complement cascade as diagnostic biomarkers are rather unsuitable, although they could provide a possibility for the determination of progression and give approaches for glaucoma therapy.

In contrast to proteins proposed as biomarkers, proteins that have a link to neuronal physiology in glaucomatous tissues are exclusively down-regulated [34,36]. These proteins, such as crystallins or neuroserpin, may help distinguish glaucoma from other neurodegenerative or different eye diseases.

### 2.5. Autoantibodies

Since the late 1990s, glaucoma has been thought to have an autoimmune component [13,14,15], manifested by measurable and decreased autoantibody titers in patients’ serum and AH, and IgG depositions found in the retina. Increased autoantibody (AAB) levels are suspected of having a neurodestructive effect, and decreased AAB levels are thought to have a reduction or loss in their protective effect [89]. Thus, proteomic analysis of AAB profiles is also a promising strategy to identify biomarkers for diagnosis, progression, or therapeutic approaches. AABs can be detected in serum samples by different methods. For example, classical detection of AABs usually takes place in several steps. First, proteins from ocular cells or animal tissue are separated using SDS gels, followed by Western blotting. The membrane is then incubated with patient serum. AAB reactivities can then be determined by mass spectrometry. With another method AMIDA for example, immunoprecipitation is used for separation with subsequent MS analysis [90]. This is often validated by microarray analyses in which recombinant antigens are incubated with serum, but also using phage displays to detect AAB reactivities against synthesized proteins are prominent [91]. The first up-regulated AABs identified in the past were AABs against heat shock proteins [92], gamma-enolase [93], alpha-fodrin [94], and myelin basic protein [95]. There are many studies about AABs against heat shock proteins, their overrepresentation in glaucoma serum samples and the destructive effect on retinal cells, well-reviewed in Tsai et al., 2019 [96].

The first AAB we want to discuss is αB-crystallin. As mentioned above, the protein level is down-regulated in different tissues of glaucoma patients, while the AAB was found to be increased in serum and AH samples of POAG patients in comparison to healthy subjects measured by microarray and Western Blot (Table 2) [97,98]. There was also a significant increment of the AABs in the serum of NTG patients in comparison to both healthy subjects and POAG patients [98]. In contrast, in the AH of NTG patients, a down-regulation of AABs against αB-crystallin was found [99]. The controversial overregulation of AABs against αB-crystallin in the serum of NTG patients, which is even significantly higher than in POAG patients, compared with the down-regulation in AH of NTG patients, is possibly explained by the methodology. The AH of glaucoma patients must be compared to cataract patients due to the invasive sampling and cataract patients have significantly higher AAB reactivities than glaucoma patients, especially after cataract surgery [100]. In addition, antibodies against αB-crystallin were detected in cerebrospinal fluid from multiple sclerosis patients and sera from mice of an experimental autoimmune encephalitis model. This underscores the role of crystallins in autoimmune diseases. Hence, up-regulated AABs against crystallins are not unique in glaucoma patients and were also measurable in the serum of SLE patients [101], and in serum of a small number of AMD patients [102].

Additionally, up-regulation of AABs compared with down-regulation of protein levels correlates very well, further implicating the protective and chaperone-like effect of αB-crystallin protein, or, conversely, underscoring the hypothesis that overregulated autoantibodies have a neurodestructive character and make the crystallins a promising tool for therapeutic approaches.

Another protein profile correlating with the AAB profile in glaucoma patients is the glial fibrillary acidic protein (GFAP), an intermediate filament primarily expressed in the retina in astrocytes and Müller cells. While the protein is highly up-regulated in glaucoma patients due to microglia activation, the AABs against GFAP were found to be down-regulated in serum and AH samples of POAG patients in comparison to healthy subjects [95,97]. Protein up-regulation of GFAP was also found in other neurological diseases such as multiple sclerosis [103], SLE [104], or epilepsy [105], indicating a link between immunological processes and neurological diseases. Increased AAB reactivities against GFAP could be found in the serum of autism and AMD patients [102,106]. The presence of GFAP AABs is also described for a novel astroglial autoimmune disorder characterized by CNS inflammation [107], but these studies so far did not investigate whether the reactivities are up- or down-regulated.

This might classify down-regulation of GFAP AABs as potential diagnostic marker for glaucoma, possibly in combination with further AAB biomarkers. Whether a differentiation to other neurological diseases can be achieved based on the measured autoantibody titers needs to be further validated. Furthermore, besides its diagnostic property, GFAP seems to be an interesting therapeutic target due to its dysregulation in many diseases.

Furthermore, differential AAB reactivity against vimentin (VIM), another intermediate filament, was found in serum samples from glaucoma patients compared with healthy subjects. Inconsistently, while down-regulated reactivities of serum samples against bovine optic nerve antigens were measured using Western blotting [95], increased AAB reactivities were measured in serum samples against porcine TM antigens by mass spectrometry, validated by microarray analysis [108].

The possibility for this discrepancy may be either the antigen or serum source, or the methodological analysis, which will be discussed in the next section.

In addition, the second-mentioned study showed that AAB reactivities against the proteins 60 kDa heat shock protein (HSPD1/HSP60), previously identified by Wax et al., 1998 [109] in NTG patients, caldesmon (CALD1), voltage-dependent anion-selective channel protein 2 (VDAC2), and phosphoglycerate mutase 1 (PGAM1) were also increased in serum samples from glaucoma patients. All markers, except HSPD1, were suitable to distinguish mild forms of glaucoma from healthy subjects [108]. Specifically, these five AABs show that individually they have weak diagnostic potential because they are not glaucoma specific but training of artificial neural networks with a panel of these potential five biomarkers showed that glaucoma patients could be classified with a sensitivity of 81% and a specificity of 93%.

For AAB reactivity against an additional protein, HIST1A4, the same issue as for VIM is evident. It was also identified with increased AAB reactivities in serum samples measured against human retinal antigens [110], whereas incubation of serum samples against human TM cell line (HTM) and glaucomatous TM cell line (GTM) antigens showed significantly decreased reactivities [111].

**Figure 2:**
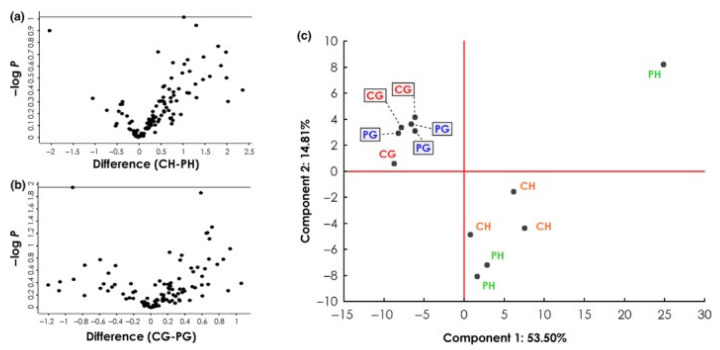
Analysis of autoantigen abundance in different experiment groups (IDs: PH, CH, PG, CG). (**a**,**b**) Volcano plots showing negative log10-transformed *p*-values (*t*-test with permutation-based FDR to correct for multiple testing; y-axis) against the differences of the means (log2; x-axis) of two groups. (**a**) Comparison of antigens from healthy TM cell lysates captured by antibodies from control (CH; *n* = 3) and POAG sera (PH; *n* = 3). (**b**) Comparison of antigens from glaucomatous TM cell lysates captured by antibodies from control (CG; *n* = 3) and POAG sera (PG; *n* = 3). (**c**) Principal component analysis, including all four experiment groups, reveals that most of the variance between the groups can be attributed to the cell line used rather than the antibody source. Published by Beutgen et al 2020 [111]. No changes were made. This work is open access. The copyright is available online: http://creativecommons.org/licenses/by/4.0/ (accessed on 7 December 2021).

These discrepancies suggest two main conclusions: (1) the antigen source has an impact on the AAB reactivity, or (2) patient samples differ considerably. Although no information was provided on the progression of the glaucoma patients, the study group sizes, as well as the mean age of the glaucoma patients, were almost the same. Therefore, an influence coming from the serum samples should be unlikely. This was confirmed by a cluster analysis published by Beutgen et al., 2020, which showed that the largest variance between the studied groups arises from the use of different antigen sources, HTM vs. GTM cell line (Figure 2). This is transferable to the studies that analyzed AABs against VIM, with the use of bovine vs. porcine antigens.

Another study identified agonistic AABs against the beta-2 adrenergic receptor (β2AR) via bioassay in sera from POAG and OHT patients [112]. For this purpose, cardiomyocytes expressing β2ARs were incubated with patient sera and subsequently, the activation rate (beating rate) of the receptors was analyzed. β2AR blockers such as Timolol are known to lower IOP by inhibiting the rate of AH formation [113]. In a subsequent study, the group demonstrated that AABs against β2AR could be detected exclusively in patients and not in healthy volunteers [114]. Sera from POAG patients (in 82% AABs detectable), patients with POAG but no perimetric deficits (in 82% AABs detectable), patients with secondary open-angle glaucoma (in 92% AABs detectable) and OHT patients (in 73% AABs detectable) were analyzed. The authors suggested that since β2ARs are expressed in TM, ciliary body, human optic nerve, and microvessels, the receptors, in addition to AH drainage regulation, may also affect microcirculation and neuron degeneration, both of which are early factors in the etiopathogenesis of glaucoma. In addition to ocular diseases with increased IOP, β2AR AABs were detected in the serum of asthma patients [115], and in sera of 59% AD patients [116]. Thus, despite Hohberger et al., 2019 [114] suggesting AABs against β2AR as a diagnostic marker for IOP-dependent ocular diseases, AABs alone are neither specific for ocular diseases nor do they provide information about progression. Possibly, a study of sera from NTG patients could provide information on whether β2AR AABs correlate exclusively with an increase in IOP or whether other factors of glaucoma pathogenesis influence their formation. Nevertheless, β2AR AABs offer a target for therapeutic treatment to reduce IOP as well as to possibly influence microcirculation or to investigate additional effects on neurons.

However, inconsistent measurements of serum samples, the problem of antigen source used for AAB detection, and the specificity of biomarkers, show that further knowledge of the distribution of autoantibodies in the population is important to validate.

**Figure 3:**
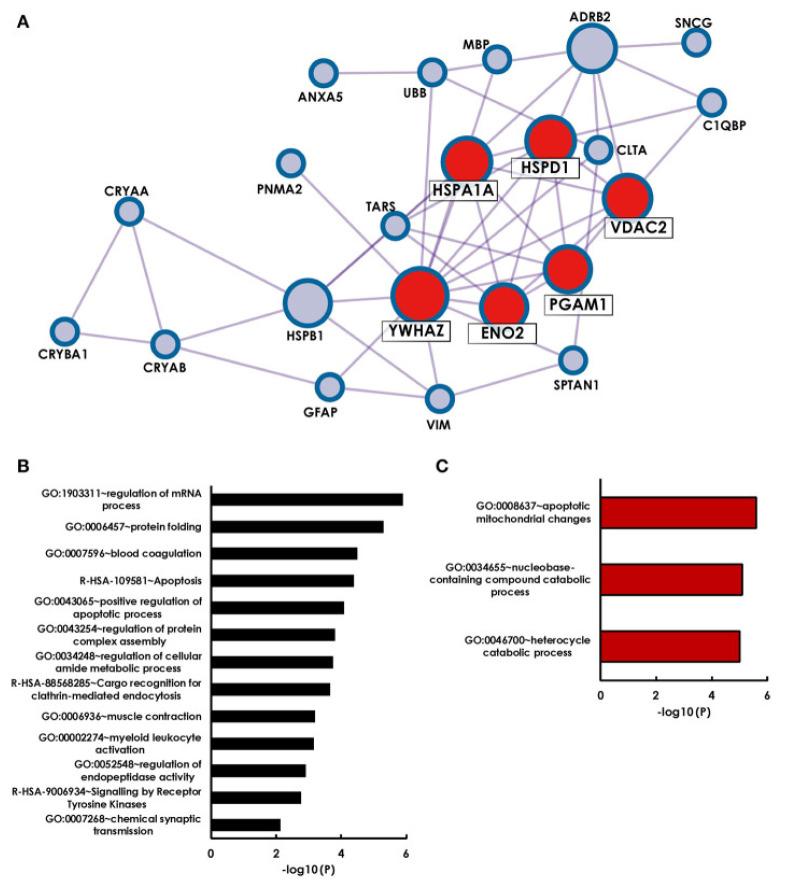
Holistic bioinformatics analysis of previously established glaucoma-related autoantigens. (**A**) Protein–protein interaction network. Twenty-two of the twenty-eight glaucoma-related antigens have at least one interaction partner among each other. Especially strong interactions were observed for six antigens (HSPA1A, HSPD1, YWHAZ, VDAC2, PGAM1, ENO2), that were identified by the Metaspcape algorithm MCODE (marked with red spots). (**B**) GO analysis of 28 glaucoma-related antigens. Shown are significantly enriched GO terms of biological processes and reactome gene sets. (**C**) GO analysis of MCODE cluster antigens only. Published in Beutgen et al. 2021 [117]. This work is open access. The copyright is available online: http://creativecommons.org/licenses/by/4.0/ (accessed on 7 December 2021).

A second important aspect is to interpret already existing data and to put it in context with new results. In a recent study, antigens that were identified as targets for glaucoma-related autoimmunity were summarized by a literature search and bioinformatical analysis with the Metascape algorithm MCODE revealed that 6 of 28 previously identified antigens form a strong network (HSPA1A, HSPD1, YWHAZ, ENO2, PGAM1 and VDAC2) [117] (Figure 3). Surprisingly, additional observation showed that four cluster antigens are localized to the myelin sheath. Retinal ganglion cells themselves, are unmyelinated until they exit the optic nerve through the lamina cribrosa. The authors themselves suggested that antibody mediated damage to the myelin sheath of the optic nerve beyond the lamina cribrosa could cause neuronal damage in glaucoma. This study clearly shows that new data should always be compared with existing data in order not only to search for biomarkers but also to provide combined information about pathogenetic mechanisms.

However, the studies on autoantibodies show that they have the potential to serve as diagnostic markers additionally to proteins, as they are significantly different between healthy and diseased patients. The loss of certain autoantibodies seems to be associated with neurological damage, suggesting a loss of natural protective autoimmunity. An advantage to the protein-based studies mentioned above is that all measurements were performed with serum samples collected in a non-invasive manner. Additionally, since it was shown that autoantibody reactivity between blood samples and AH samples correlate well in both glaucoma patients and healthy subjects [97], this indicates a high specificity of disease-related autoantibody changes.

Another advantage of detecting autoantibodies compared to protein-based measurements is that altered composition of these may be possible prior to loss of ganglion cells and thus before visual field defects.

## 3. Therapeutical Approaches for Future Glaucoma Therapy

In the first part of this review, it became clear that an effective screening of molecular changes in eye tissues such retina, aqueous humor and vitreous or in serum samples can also provide information to find possible approaches for new therapeutic options. In this context, some selected targets and their therapeutic potential will be evaluated in the next part of the review.

### 3.1. Crystallins

Maintaining homeostasis in tissues and organs as well as cells is one of the most important functions for cell survival. Under stress conditions, increased protein production occurs, which in turn can lead to an increased rate of misfolding [118]. Chaperones are proteins that promote and mediate the proper folding of proteins by transiently binding to the polypeptide chains, allowing the stepwise folding of regions not held in the unfolded state by the chaperones [119]. Special chaperones are expressed in an initial response immediately after a stressful stimulus. These proteins, called heat shock proteins (HSPs), are divided into different groups, named according to their molecular weight, for example, HSP27 and HSP60. In glaucoma, AAB reactivities against HSPs in sera and patients were found in very early studies and were associated with increased neurodegeneration in the retina [95]. This underlines the importance of chaperones in glaucoma. Another group of small HSPs specifically found in the eye is the crystallins [120]. That the change in the expression of different crystallins is a very early response of retinal cells to stress was shown by an mRNA-based analysis in consequence of a chronic IOP increase [121]. The mRNA levels of especially βB2-crystallin and αA-crystallin but also αB-crystallin were significantly decreased eight days after IOP elevation, but after five weeks the mRNA increased to baseline levels. In some cell culture experiments, α-crystallin was added to cells stressed with different stressors, and neuroprotective properties of crystallins were discovered and important signaling pathways were searched. Thus, rat primary astrocytes stressed with staurosporine and C2-ceramide were protected by the addition of α-crystallins, through MAPK signaling pathway activation by αA-crystallin and PI3K/Akt signaling pathway activation by αB-crystallin [122]. Furthermore, this experiment demonstrates that the addition of crystallins contributed to blocking the release of ROS. In retinal neurons, hypoxia-induced expression of caspase 3 was shown to be inhibited by increasing endogenous expression of α-crystallin [123]. Thus, the authors suspected a neuroprotective effect of overexpressed α-crystallin. Similar protective effects were observed in an animal model of induced inflammation [124]. Here, treatment with α-crystallin was shown to reduce inflammation by preventing the activation of GFAP and NFκB_p65_ in the neocortex of mice demonstrated by immunohistochemistry. Furthermore, α-crystallin, reduced inflammation-induced intracellular calcium levels. In further experiments in this study, α-crystallin was shown to have a scavenging ability toward sulfur and nitrogen oxides. Inhibition of astrocyte activation was also demonstrated in an optic nerve crush model by intravitreal application of αA-crystallin [125]. The mechanism of neuroprotective action is not fully understood; however, recent studies showed that phosphorylation of tyrosine 148 mediates protective properties on αA-crystallin in experimental diabetic models [126]. This was confirmed in another study in which Müller glial cells (MGCs) were isolated from HspB4/αA-crystallin knockout mice and subsequently cultured [127]. Cells were then transfected with a phospho-mimetic (T148D) or a non-phosphorylatable αA-crystallin variant (T148A) or a wild-type (WT) variant of HspB4/αA-crystallin, respectively. After stress induction by serum starvation or the addition of glucose and TNFα, WT or T148D overexpression showed that the expression of the proinflammatory cytokines IL-6, IL-1, and IL-18, as well as NLRP3 and NFκB, was significantly lower compared with these expressions in cells expressing the T148A variant. This indicates that phosphorylation of tyrosine 148 plays a crucial role in the neuroprotective effect of crystallins in experimental diabetic models.

In addition to αA-crystallin, some studies have also shown that αB-crystallin have a neuroprotective effect in models of neurodegenerative diseases such as rhegmatogenous retinal detachment [128] or Huntington’s disease [129]. In endotoxin-induced uveitis, αB-crystallin appeared to reduce retinal inflammation by inhibiting microglial activation and autophagy [130]. Cell culture experiments showed that phosphorylation of Serins 45 and 59 of αB-crystallin determined p38 activation and thereby protected astrocytes isolated from rat brain previously stressed with staurosporine or C2-ceramide [131]. The protective function of phosphorylation of Serins 45 and 59 was also described for rat hippocampal neurons [132]. Another mechanism of astrocyte protection in the central nervous system was described by gain or loss of function studies [133]. Here, it was shown that knocking out the dopamine D2 receptor (DRD) in astrocytes made them more susceptible to the neurotoxin 1-methyl- 4-phenyl-1,2,3,6-tetrahydropyridine (MPTP) and increased the inflammatory response. This effect was shown to be dependent on the expression level of αB-crystallin. The authors concluded that αB-crystallin may represent a new therapeutic option for the astrocyte-mediated innate immune response in the central nervous system. Supporting this, another study showed that αB-crystallin may play a protective and therapeutic role in autoimmune demyelination [47]. In that study, knockout of αB-crystallin in the EAE model showed increased Th1 and Th17 cytokine release from T cells and macrophages and more intense CNS inflammation compared with wild-type. As described above, AAB against crystallins is elevated, therefore crystallin application has a good therapeutic potential to overcome autoimmunity [134]. In terms of therapeutic potential, a clinical phase IIa trial showed that low-dose use of alpha B crystallin reduced the number and volume of MRI-active lesions in relapsing-remitting multiple scleroisis by 76% [135]. Therefore, also based on the experimental studies, the application of crystallins could be a therapeutic approach to glaucoma therapy.

The role of βB2-crystallin in the context of neuroprotection in retinal diseases has received little attention. Nevertheless, one study showed that βB2 mutations are critically involved in cataract formation in patients and that these patients also developed glaucoma more frequently [136]. The authors described a mechanism in which gene conversion leads to amino acid conversion, which changes the solubility and subcellular localization of the protein. This is consistent with a study describing in cell culture experiments that αB-crystallin can bind to a myocilin variant, which is closely associated with glaucoma development, thereby promoting degradation of this complex [137].

The neuroprotective potential of βB2-crystallin was demonstrated in an experimental glaucoma animal model, in which intravitreal injection of this protein increased retinal RGC survival and nerve fiber layer thickness compared with controls [138].

### 3.2. GFAP

A major risk factor of glaucoma disease is an elevated IOP, which can be severe at certain points (acute glaucoma) or chronically elevated as in primary open angle glaucoma (POAG), causing mechanical pressure on the retinal tissue. This could have an impact on the cytoskeleton of the cells of the retina. The cytoskeleton of neuronal cells consists, among others, of intermediate filaments, which are divided into six different types (type I–VI).

Lamins, type V intermediate filaments are mainly found in cell nuclei [139], while keratins, type I and II intermediate filaments are found in epithelial cells, and neurons have type VI intermediate filaments, the so-called neurofilaments, which also include nestins [140].

Glial cells, i.e., astrocytes and Müller cells possess type III intermediate filaments, such as vimentin and GFAP [141,142,143]. These two proteins contribute to retinal intactness.

However, these proteins not only serve a structuring function but specifically GFAP is also crucial for maintaining homeostasis and internal tissue repair [144]. In the central nervous system, for example, specialized cells, called astrocytes, undergo GFAP-induced structural modulation, and become capable of migration after activation. Migrating astrocytes surround the inflammatory hotspot and separate it from the undamaged tissue, which is initially a neuroprotective effect. This formation process is called lesion formation or reactive gliosis [145].

In addition to this neuroprotective role, it seems increasingly clear from recent studies that GFAP also mediates a neurodegenerative effect on various cells in the retina. In experimental animal models characteristic of acute angle closure glaucoma [146] and other OHT models, like MB model [147,148], EVO model [149], or genetic model of POAG [150], each showed increased expression of GFAP. This in turn results in a lower proliferation of astrocytes in the optic nerve head immediately after IOP elevation, but especially in a disruption of the connections of astrocytes to the basement membrane and an increase of the extracellular space in the optic nerve head. It was observed that axonal transport and thereby homeostasis was disrupted as a result [151]. The activation of JAK-STAT signaling pathway has been identified as a major pathway for the increase of GFAP expression in the optic nerve head, which is activated at an early stage of optic nerve damage by IOP increase [152]. Sin3A and MeCP2 form a complex to inhibit a promotor responsible for GFAP gene transcription. If the complex dissolves, STAT 3 is activated and recruits the CREB binding protein CBP300 to exon1 of the GFAP promoter and activates GFAP gene transcription. In addition, this process modulates chromatin and inhibits astrocyte differentiation and neuronal plasticity [153,154]. GFAP activates not only astrocytes but also microglia. It was shown in an experimental animal model that the level of activation of microglia correlated with the level of CD200 protein [155]. CD200 expression in turn correlated with the expression of GFAP, CD45, OX42, and OX41 in the optic nerve head. In a culture of activated retinal Müller cells, incubation with TNF-α showed increased expression of GFAP, iNOS, IL6, and further cytokines were partially regulated by the NFκB pathway [156]. This was confirmed in a co-culture system of retinal ganglion cells and glial cells exposed to hydrostatic pressure. In this study, TNFα was shown to be produced and released by the reactivated glial cells. TNF and nitric oxide inhibitors reduced apoptosis of RGCs [54].

Potential therapeutic approaches using GFAP as a target protein are rare. In a model of acute as well as a model of chronic IOP elevation, a potassium channel opener showed a protective effect on RGC density in the retina by reducing Müller cell activity, as evidenced by lower GFAP expression [157]. A diet containing coenzyme Q_10_ showed an inhibitory effect on glutamate excitotoxicity by reduction of GFAP expression [158]. Rosuvastatin, a drug used as a cholesterol-lowering agent, showed increased survival of RGCs after oral administration in an experimental animal model of IOP elevation using episcleral vein occlusion. This effect was in line with a reduction of the retinal GFAP immunoreactivity of the Rosuvastatin treated animals compared to control animals [159]. These studies indicate that reduction of GFAP through other targets in retinal tissue increases cell survival, which in turn favors targeting GFAP directly. In a gene therapy approach, BDNF was continuously expressed in a model of transient IOP elevation. This increased the BDNF level in the retina, thereby reducing retinal stress and GFAP expression, which in turn resulted in enhanced survival of RGCs [160]. Peptide-based inhibition of Fas receptor also prevented axon degeneration and RGC death in a glaucoma model [161]. Naturally occurring substances such as grapeseed or the anti-inflammatory agent Wogonin also reduced neuroinflammation, as well as GFAP expression, ensuring higher survival of RGCs [162,163]. These therapeutic approaches do not target GFAP directly but modulate structures or signaling pathways associated with GFAP expression and thereby activation of astrocytes or microglia. Mainly, increased GFAP expression, which was observed in astrocytes or microglia among others, has been associated with increased intraocular pressure [148,149]. IOP up-regulation not only increased GFAP expression but also increased expression of MHC II molecules in microglia of OHT and contralateral eyes [10]. These molecules can present antigens and autoantigens to Müller cells, resulting in the recruitment of immune cells or antibodies. Interestingly, lower AAB levels against GFAP were detected in sera from POAG patients compared to sera from healthy subjects [88]. According to a hypothesis of AAB profiles, it has been shown that increased AAB reactivities, for example against HSP27, can have a neurodegenerative effect and, in contrast, down-regulated AAB reactivities can indicate a neuroprotective potential of the AABs, which is possibly lost by down-regulation. In cell culture studies, incubation with GFAP antibody reduced the loss of RGCs and decreased the level of reactive oxygen species (ROS) in H_2_O_2_-and glutamate stress-induced cells of a neuroretinal cell line [164]. The authors could show in this study that targeting of GFAP reduced the expression of actin regulating proteins like Profilin, Cofilin, and actin regulated protein 2/3. Furthermore, they could show an up-regulation of actin stabilizing proteins, which leads to the conclusion that the antibody treatment reduces oxidative stress by altering the protein expression of actin cytoskeleton-associated proteins. This was confirmed in an ex vivo model of the porcine retina in which glaucoma-like stress induction occurs through optic nerve separation [165]. Subsequent mass spectrometric analysis of porcine retina revealed a significant down-regulation of GFAP. In these two studies, GFAP was directly used as a therapeutic target, which influenced the GFAP expression itself as well as downstream signaling pathways like the mitochondrial apoptosis pathway, highlighting the potential of GFAP not only as a marker of reactive gliosis but also as a potential therapeutic target.

### 3.3. The Complement System

**Figure 4:**
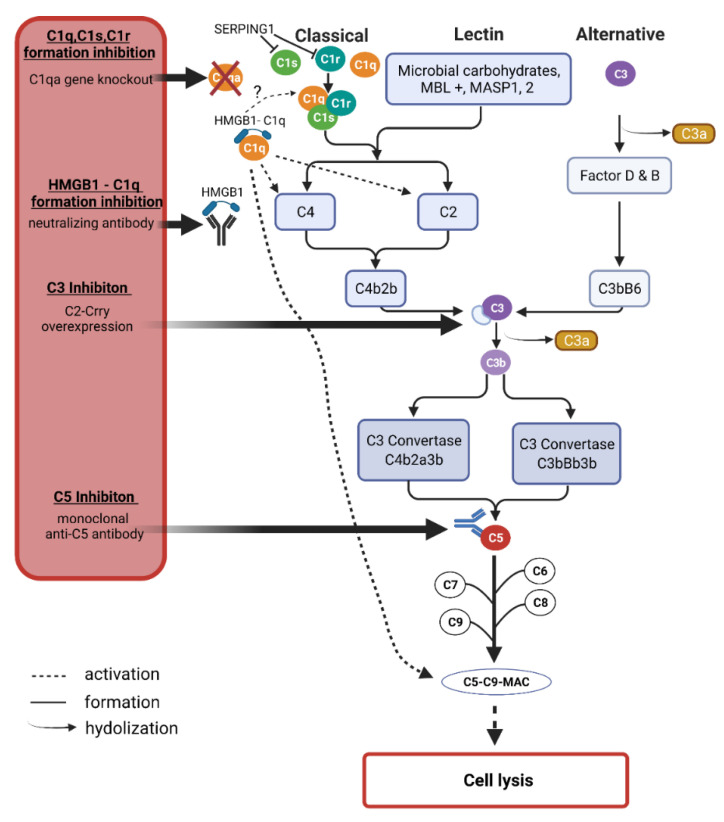
The complement system cascades and possible intervention options. The complement system is activated by the classical, the lectin, or the alternative pathway. All pathways have in common that they lead to the formation of C3 and C5, which in turn leads to the emergence of the membrane attack complex (MAC), which forms a pore in cell membranes, leading to the lysis of the affected cell. C3 is hydrolyzed to C3b and C3a. C3 and C5 represent opportunities to inhibit MAC formation. C3 can be inhibited by overexpression of the specifically designed protein C2-Crry. C5 can be inhibited by monoclonal antibodies. In addition to the universal proteins C3 and C5, C1q also offers the possibility for therapeutic intervention. Gene knockout of C1qa leads to reduced MAC formation. SERPING1, in turn, can inhibit C1r and C1s in a cellular naturally occurring process. The complement system is activated by several stimuli, one being the formation of the HMGB1-C1q complex which initiates the formation of C4, C2, and the MAC. This can be prevented by a neutralizing anti-HMGB1 antibody. The graphic was created by BioRender.com. The copyright was gained from Biorender.com (accessed·on 31 October 2021).

The complement system is primarily involved in the defense against invading pathogens along with other cell types such as microglia and dendritic cells in the clearance of these pathogens [166]. As mentioned, the complement system is activated through three different pathways, the lectin, the classical, or the alternative pathway (Figure 4). The lectin pathway is activated by the protein mannose-binding lectin (MBL) recognizing sugar residues on the surfaces of pathogens and forming complexes with mannose-specific serine proteases 1 and 2 (MASP1 and 2), which activate the further cascade and eventually form the membrane attack complex (MAC) that initiates pore formation in cell walls and thus cell death and degradation [167]. In the classical pathway, C1q binds to antibodies or antigen-antibody complexes, among others, which in turn activates C3 to then activate C3b and C5 with the goal of MAC formation, as in the lectin pathway [168]. The alternative pathway is spontaneously activated by hydrolysis of C3 to C3b and C3a [169].

Under physiological conditions, the complement system is also activated at a low level to maintain microenvironmental homeostasis the so-called parainflammation [170]. However, this activation in retinal tissue is hypothesized to elevate with increasing age, thus the expression of complement-associated proteins is increased even under non-inflammatory conditions [171]. It was suggested that there is a progression from low-level activation to high chronic activation of the complement system in glaucoma patients [172]. Given their universality of activation across all three pathways, C3 and C5 are promising candidates in identifying potential targets of therapeutic intervention. The complement component C1 seems to be an attractive target, as especially the proteins of the classical pathway were found to be increased while the major complement regulator, complement factor H was decreased by oxidative stress in vitro [54].

The temporal component of complement activation was further investigated in an experimental animal model of ocular hypertension by saline injection into the episcleral veins of rats. The mRNA levels of several proteins were examined after eight days as well as after five weeks [121]. Thereby, C1qb, C1s, and C1r, as well as C3, were noticeably increased after eight days. After five weeks, these trends were even more evident. These findings were confirmed in a pressure-independent model, using optic nerve transection. Of the total 25 mRNAs tested, 24 were confirmed with the same trends by the second model. This indicates that activation of the complement system may well be long-lasting. As described in the section “diagnosis & screening” of this review, C3 and especially the C3a/C3 ratio could serve as a marker of the disease state in glaucoma progression. Concerning a possible therapeutic approach using gene therapy, a C3 inhibitor was overexpressed in the chronic glaucoma animal model of DBA/2J using an adenovirus [173]. Here, sustained overexpression of the inhibitor resulted in the reduced deposition of complement components, particularly C3d in retinal ganglion cells. This regulates neurodegeneration and slows the progression of RGC loss despite continued IOP elevation.

In an experimental autoimmune glaucoma model (EAG) IOP-independent immunization with human optic nerve homogenate was able to induce RGC loss within 6 weeks, associated with functional retinal impairment [56]. In this study, the potential of two anti-C5 monoclonal antibodies was investigated. Pre-application of the antibody two weeks before immunization demonstrated that while both antibodies reduced the loss of RGCs, only one clone also possessed positive effects on retinal functionality. Furthermore, the number of activated microglia or the expression levels of proinflammatory cytokines such as IL-1β was examined.

Interestingly, the number of activated microglia was increased in the groups with the injection of the C5 antibody. The authors concluded that this was due to the intravitreal injection itself, as similar effects were already observed in previous studies [174,175]. When considering the mechanism, it stood to reason for the authors that C5 inhibition prevents the formation of MAC, thereby decreasing the rate of apoptotic RGCs. Since the number of caspase 3^+^ cells was approximately the same in all groups, it can be assumed that apoptosis pathways other than the caspase 3 pathway were modulated by C5 inhibition in this model. This indicates that the complement system can influence the cell death of RGCs through different pathways.

The classical pathway of complement activation is investigated concerning a possible target for neuroprotection. To this end, it was shown in the DBA/2J glaucoma animal model that a gene knockout of C1qa had a neuroprotective effect on both retinal RGCs and optic nerve damage and therefore, according to the authors, represents a promising target for inhibition of the classical pathway [176].

This shows that not only the C3-dependent pathway but also the C3-independent pathway should be considered in therapeutic modulation of the complement system, especially since not only C3 but also C1q was up-regulated in early stages of glaucoma in different cell types such as retinal ganglion cells as well as in the optic nerve head [177]. C1q can bind pathogens or even apoptotic cells, which triggers a signal for phagocytosis and can induce a further inflammatory cascade by modulating the Wnt pathway or releasing cytokines [178]. In an autoimmune glaucoma animal model, IOP independent immunization with S100B activated both the complement system and NFκB signaling pathway [56]. Therefore, intervention at the complement system appears to be a promising approach for a therapeutic option. The aim of an intervention in the complement system should be to revise the differentially expressed proteins to a normal level. Complete inhibition could have negative effects since the parainflammation could be disturbed, which contributes to maintaining retinal homeostasis.

Furthermore, the intervention should target the affected region as precisely as possible. The blood-retina barrier (BRB) prevents the systemic distribution of the therapeutic agent and is therefore limited to the eye when applied intravitreally.

As described previously, experimental studies in glaucomatous disease showed that targeting C1, C3, or C5 may have neuroprotective effects. Drugs targeting the complement system, such as monoclonal AB against C3 or C5, have been tested in several clinical trials (phase II and III) for various diseases, including age-related macular degeneration [179,180]. To our knowledge, clinical trials testing these drugs in glaucoma patients are not listed in *ClinicalTrials.gov*. However, as their targets are often identical to the listed clinical trials, this could be a potential approach for translational research. At the same time, however, a key challenge emerges. Since C3 or C5 are targets for a wide variety of diseases, indicating that they are not disease-specific or even glaucoma-specific. Nevertheless, a translational approach could be promising, as the multifactorial nature of glaucoma treatment could provide a basis for combination therapy with other promising drugs, for example, other monoclonal antibodies.

However, further therapeutic options need to address cellular levels other than the complement system, as glaucoma represents a multifactorial disease.

### 3.4. High-Mobility Group Protein 1 (HMGB1)

As described above, the components of the complement system are differentially regulated, which in turn mediate the further inflammatory response through, among others, damage-associated molecular patterns (DAMPS), i.e., endogenous danger molecules that are released from damaged or dying cells. A key molecule that also interacts directly with the complement system and was up-regulated in glaucoma patients is the high mobility group protein B1 (HMGB1) [35]. HMGB1 belongs to a large family of non-histone binding proteins. This group includes the proteins HMGB1, HMGA1, and HMGC. HMGB1 is expressed in all retinal neurons with different isoforms [181]. The localization of the protein determines its function. Under non-stress conditions, it is mainly found in the nucleus where it can modulate transcription by binding to transcription factors [182]. Under stress conditions, the protein can be translocated into the cytoplasm and is accessible for further binding partners. HMGB1, for example, was identified as a new Beclin1 binding protein and may thus be involved in autophagy [183]. Furthermore, it is found in mitochondria and the extracellular space, where it serves as a ligand for a wide variety of receptors that can, for example, transmit an inflammatory response [184].

The protein is also involved in the organization of inflammatory responses of different immune cells. HMGB1 is actively or passively secreted by immune cells such as macrophages, astrocytes, and microglial cells as well as by damaged, necrotic, or dying neurons [185]. When expressed on the surface, HMGB1 is an activator of the innate immune response [186]. As described above, the complement system plays a major role in the innate immune response. In this regard, the C5-C5a anaphylatoxin chemotactic receptor (C5aR) interaction complex is described in the literature to be critically involved in the organization of inflammatory responses [187]. This is predicted for NLRP3-inflammasome-associated diseases. Yu and others demonstrated that deficiency of C5aR restricted NLRP3 inflammasome activation, which triggered the release of cytokines, and reduced HMGB1 release in vivo and in vitro [188]. According to the authors, activation of the inflammasome by C5aR2 occurs via amplification of double-stranded RNA which in turn results in protein kinase R and subsequently NRLP3 activation in macrophages. In a sublethal endotoxemia model, C5 was shown to cause significantly increased LPS-induced production of the proinflammatory cytokine IL-1β [188]. This effect could again be ameliorated in C5aR1 deficient mice. A differential effect could be determined for C5a in this model to NLRP3 activation. In monocytes, C5 was able to activate the NRLP3 inflammasome via Toll-like receptor 4, whereas in macrophages C5 exerted more of a suppressive effect on the NRLP3 inflammasome [187].

This seems to contrast with the previous studies, yet in summary, the authors concluded that C5aR deficiency attenuates the IL-1β response to LPS-induced stress in vivo and C5a overall enhances the physiological inflammasome response. In addition to its effect on C3 or C5, Kim and others demonstrated that HMGB1 can activate the classical complement system signaling pathway [189]. In human plasma, MAC could be detected after HMGB1 addition. This complex accumulated on vessels where HMGB1 also accumulated. This effect was suppressed by the neutralization of HMGB1. Thus, the authors concluded that HMGB1 triggers the classical signaling pathway of the complement system under ischemic conditions and the onset of cell necrosis, thereby inducing sterile inflammation in an Acetaminophen-induced hepatotoxicity model [189]. An extended immune response by retinal tissue-infiltrating T cells does not usually occur under physiologic conditions if BRB intactness is maintained [190]. Antibody deposition in retinal tissue was detected in glaucoma [191,192,193], suggesting that the BRB increases its permeability under pathological conditions. That is in line with the results published by Chen et al., 2018 [194], that elevated IOP can serve as a trigger for T cell infiltration, possibly due to decreased BRB integrity. In animal models of diabetic retinopathy, a regulatory function of HMGB1 on the BRB is described [190]. In diabetic eyes, in which there was damage to the BRB, HMGB1 and the receptor for advanced glycation (RAGE) were found to be up-regulated. Brain-derived neurotrophic factor (BDNF) was found down-regulated in the serum of diabetic patients, in contrast to HMGB1 and soluble RAGE, among others, which were found up-regulated [195]. HMGB1 and BDNF levels were inversely correlated in serum samples in this study. In an experimental diabetic animal model, HMGB1 was injected intravitreally into rats, with the result that HMGB1, thiobarbituric acids (TBARS), and cleaved caspase 3 were increased, and BDNF and synaptophysin were significantly down-regulated in these retinas [195]. In glaucoma, BDNF has already been shown to modulate neuroprotection, highlighting the potential of HMGB1 as a target for possible intervention in glaucoma [196,197].

That HMGB1 also has an impact on specialized cells such as Müller cells was shown Here, human retinal Müller glial cells were stimulated with HMGB1 or high glucose to simulate the situation in the diabetic retina. It was shown that in these cells HMGB1 modulated STAT-3 expression and promoted translocation to the nucleus.

This highlights the diversity that targeting HMGB1 could mean in different diseases. However, HMGB1 can act not only intracellularly but also via various chemokine receptors. Together with stromal cell-derived factor 1 (CXCL12), HMGB1 mediates the recruitment of inflammatory cells to the site of inflammation in the CNS [198]. This receptor complex influences blood–brain barrier integrity and ensures that peripheral immune cells can enter the CNS. The molecule CXCR7, an atypical chemokine receptor 3, was shown to be an inhibitor of the HMGB1-CXCL12 complex that was able to reduce HMGB1-induced neuroinflammation [199].

That HMGB1 with the receptors CXCL12 and CXCR4 is also involved in autoimmune diseases has already been reported [200]. In an experimental model of chronic autoimmune uveitis, the HMGB1-CXCL12 complex was shown to promote the entry of inflammatory cells into retinal tissues [201]. In bone marrow-derived macrophages, CXCL12 level was shown to be dependent on HMGB1 level [202].

In SLE patients and due to brain injuries of chronically HIV-infected patients, autoantibodies against HMGB1 were detected [203,204]. Furthermore, one study demonstrated that HMGB1 may be involved in initiating the production of autoantibodies [205]. It has been described several times that glaucoma also has an autoimmune component that may contribute to disease progression [206,207,208], again showing that modulation of HMGB1 protein may be a promising approach for glaucoma disease therapy.

The proinflammatory role of HMGB1 was described in a study in which mouse retinal photoreceptor-derived cells (661W) and retinal explants were exposed to elevated pressure in a pressure chamber [209]. Depending on the pressure elevation, an up-regulation of HMGB1 and the associated receptors RAGE, TLR-2, TLR-4, TNFα, and other proteins, that were more strongly expressed in apoptosis signaling pathways, was observed. This was also demonstrated in a pressure-independent manner by incubation with exogenous HMGB1 (rhHMGB-1) in both 661W cells and retinal explants.

In the retina, the effect of HMGB1 and NFκB was investigated by pressure-independent NMDA-induced stress on rats [210]. Here, intravitreal injection of NMDA induced the up-regulation of HMGB1 and NFκB.

In experimental animal models of acute glaucoma, HMGB1 was shown to force activation of NLRP3 and the caspase-8 inflammasome through the NFκB signaling pathway [211].

To date, the question of the effect of HMGB1 in a chronic glaucoma model seems open.

## 4. Conclusions

Glaucoma is generally defined as a multifactorial disease. Therefore, a single treatment may not be as promising as combined therapeutic approaches. Screening approaches in the experimental field are very useful, as they can provide insight into pathological alterations of different tissues, on the transcriptional or translational level, but also the proteomic level. Here, we reported milestones set by utilizing microarray and mass spectrometry, which significantly increases the selection of possible therapeutic targets. Although, detected differences can vary depending on the selected model. The complement system, for example, is activated in IOP-dependent and independent glaucoma animal models and has therefore become a particularly promising focus of research in recent years, even though the system is not only glaucoma-related.

More specific research approaches are provided by targeting cytoskeletal organizations such as crystallins, or autoantibody-based approaches, as shown for GFAP AABs or β2AR AABs.

It will be crucial to perform proteomic techniques with patient samples and to compare the patient profiles with the known results from basic research to develop appropriate therapies tailored to the patient. For these therapies, the starting time is essential, as immune activation can be detected even before RGC loss in the models. Therefore, screening and the precision of screening procedures are necessary for glaucoma detection and bridging the gap from the classic examinations to new therapeutic strategies.

## Figures and Tables

**Table 1:** Differentially regulated Glaucoma-related proteins identified in different eye tissues such retina, aqueous humor and vitreous, or in serum samples of glaucoma patients by different previous studies. ↓ demonstrates down-regulation of the proteins in glaucoma patients/donors, ↑ shows up-regulation, while ↔ means that no differential expression could be measured between glaucoma patients and healthy subjects. Arrows in the “Serum” column are shown in red for better visualization. The type of glaucoma induction in animal models is shown with abbreviations. TLP: trabecular photocoagulation, MB: microbead injection, EVO: episcleral vein occlusion, ONC: optic nerve crush.

Gene Name	Protein Name	Retina	Aqueous Humor	Vitreous	Serum	Animal Model
CRYAA	α-Crystallin A chain		↓ [34]	[33]		↓ TLP [38] ONC [39]
CRYAB	α-Crystallin B chain	↑ [35]		↓ [33]		↓ MB [36] EVO [37] TLP [38] ONC [39]
CRYBA1 (CRYBA3)	β-Crystallin A3	↓ [35]		↓ [33]		
CRYBA2	β-Crystallin A2			↓ [33]		↓ MB [36] EVO [33]
CRYBA4	β-Crystallin A4			↓ [33]		↓ MB EVO [33]
CRYBB1	β-Crystallin B1	↓ [35]		↓ [33]		↓ MB [36] EVO [37]
CRYBB2	β-Crystallin B2			↓ [33]		↓ MB [36] EVO [33] TLP [38] ONC [39]
CRYBB3	β-Crystallin B3			↓ [33]		↓ MB [36] EVO [37]
CRYGB	γ-Crystallin B			↓ [33]		
CRYGD	γ-Crystallin D			↓ [33]		
CRYGS	γ-Crystallin S			↓ [33]		↓ MB [36] EVO [37]
TPM1/TPM3 TPM4	Tropomyosin alpha-1 chain/alpha-3 chain/alpha-4 chain	↓ [36]				
IQGAP2	Ras GTPase-activating-like protein IQGAP2	↓ [36]				↓ MB [36] ↑ EVO [37]
AHNAK	Neuroblast differentiation-associated protein AHNAK	↓ [36]				↓ MB [36]
DKK3	Dickkopf-related protein 3		↑ [50]			
WIF1	Wnt inhibitory factor 1		↑ [50]			
SERPINF1 (PEDF)	Pigment epithelium-derived factor		↑ [50]			
SERPINA1 (AAT)	α-1-antitrypsin	↑ [35]			↑ [51]	
SERPINA3 (AACT)	α-1-antichymo- trypsin		↑ [50]			
SERPINA6 (CBG)	Corticosteroid-binding globulin		↑ [50]			
SERPINA7 (TBG)	Thyroxine-binding globulin		↑ [50]			
SERPINA8 (AGT)	Angiotensinogen		↑ [50]			
SERPINF2 (A2AP)	α-2-antiplasmin		↑ [50]			
SERPINI9 (NEUS)	Neuroserpin	↔ [52]	↓ [34]	↔ [52]		
APOA1	Apolipoprotein A-1	↑ [33]	↑ [53]	↑ [33]	↑ [51]	
APOA4	Apolipoprotein A-4		↑ [34]		↑ [51]	
APOD	Apolipoprotein D		↑ [34]			
APOE4	Apolipoprotein E4	↑ [33]	↑ [50]	↑ [33]		↓ MB [36]
C1Q	Complement C1q subcomponent	↑ [54]	↑ [50]			
C3	Complement C3	↑ [55]	↑ [50]		↑ [51]	↑ EAG [56]
C5	Complement C5	↔ [55]				↔ EAG [56]
C8	Complement component C8	↑ [54]	↑ [52]			
C9	Complement component C9	↑ [57]	↑ [52]			
VSIG4	V-set immunoglobulin domain-containing protein 4		↑ [52]			
MASP1/MASP2	Mannan-binding lectin serine protease 1/2	↑ [57]				

**Table 2:** Results of individual biomarker power discrimination obtained with Naïve Bayes algorithm in the classification of POAG, PEXG and control serum samples with newly recruited patients (*n* = 53). Reprinted from Gonzalez-Iglesias et al. 2014 [51]. No changes were made. This work is open access. The Copyright is available online: http://creativecommons.org/licenses/by/4.0/ (accessed on 7 December 2021).

Gene Name	POAG vs. PEXG vs. Control				POAG vs. Control				PEXG vs. Control				POAG vs. PEXG			
	CA ^a^	Sens. ^b^	Spec. ^c^	AUC ^d^	CA	Sens.	Spec.	AUC	CA	Sens.	Spec.	AUC	CA	Sens.	Spec.	AUC
APOA4	0.8113	1.0000	0.9118	0.9233	0.9744	1.0000	0.9500	1.0000	0.8788	0.9474	0.7857	0.8609	0.7941	0.8000	0.7857	0.8143
C3	0.6415	0.8421	0.8824	0.8607	0.8718	0.8421	0.9000	0.9763	0.7879	0.7895	0.7857	0.8910	0.6765	0.6500	0.7143	0.6786
TF	0.6792	0.8947	0.8235	0.8283	0.8718	0.9474	0.8000	0.9526	0.8485	0.9474	0.7143	0.8120	0.6765	0.6500	0.7143	0.6571
VTN	0.6038	1.0000	0.8529	0.7786	0.9231	0.9474	0.9000	0.9474	0.8182	0.8421	0.7857	0.9060	0.4706	0.6000	0.2857	0.4464
TTR	0.5849	0.8421	0.7941	0.7635	0.8718	0.8421	0.9000	0.9342	0.7576	0.8947	0.5714	0.6617	0.5588	0.7000	0.3571	0.6750
SERPINA1	0.6038	0.8421	0.7059	0.7203	0.8462	0.8947	0.8000	0.8684	0.6667	0.7368	0.5714	0.7556	0.6176	0.8000	0.3571	0.4821
FBLN1	0.6981	0.8421	0.9118	0.8380	0.8293	0.8421	0.9091	0.8886	0.9091	0.8947	0.9286	0.8797	0.6471	0.8000	0.4286	0.5821
APOA1	0.4717	0.7895	0.7647	0.6641	0.7692	0.7895	0.7500	0.8158	0.7576	0.7895	0.7143	0.7857	0.4412	0.6500	0.1429	0.3643
FCN3	0.5849	0.6842	0.7059	0.7559	0.7692	0.8421	0.7000	0.8553	0.6970	0.7368	0.6429	0.7293	0.6176	0.6000	0.6429	0.6786
CFH	0.6226	0.7895	0.8824	0.6933	0.7949	0.6842	0.9000	0.8079	0.7576	0.7895	0.7143	0.7368	0.5588	0.7000	0.3571	0.4893
ITIH4	0.3396	0.4737	0.4412	0.4590	0.4872	0.4737	0.5000	0.6158	0.5758	0.7368	0.3571	0.3647	0.5294	0.8000	0.1429	0.3679
APOL1	0.4906	0.6842	0.7059	0.5972	0.6923	0.6842	0.7000	0.7553	0.5455	0.6842	0.3571	0.6955	0.3824	0.6500	–	0.2607
ALB	0.2453	0.3158	0.5882	0.5778	0.3846	0.3158	0.4500	0.5263	0.7576	0.8421	0.6429	0.7895	0.4706	0.6000	0.2857	0.5357
SERPINC1	0.3019	0.3158	0.6765	0.4816	0.4359	0.3158	0.5500	0.3842	0.6667	0.7368	0.5714	0.6466	0.4706	0.6500	0.2143	0.4643
IGHG2	0.2453	0.2632	0.5882	0.2894	0.3333	0.2632	0.4000	0.4000	0.2727	0.4737	–	0.2068	0.3235	0.5500	–	0.2214
C4A	0.35 85	0.5789	0.6471	0.5346	0.4103	0.4737	0.3500	0.3947	0.7273	0.7895	0.6429	0.7444	0.4706	0.6000	0.2857	0.5500
APCS	0.3208	0.2632	0.5294	0.3164	0.4359	0.3158	0.5500	0.3368	0.5152	0.7368	0.2143	0.3722	0.6471	0.9000	0.2857	0.2786

^a^ CA, correct assignment. ^b^ Sens., sensitivity. ^c^ Spec., specificity. ^d^ AUC, area under the curve.

## Data Availability

We exclude this statement because we do not present data which was not yet published.
